# Behaviour support planning in residential aged care ‐ pilot to explore implementation of new legislation in Australia

**DOI:** 10.1002/alz.084214

**Published:** 2025-01-09

**Authors:** Jacqueline Wesson, Kylie Wales, Jennifer Hewitt, Theresa M Flavin, Meredith D Gresham, Amy Tan, Henry Brodaty

**Affiliations:** ^1^ University of Sydney, Camperdown, NSW Australia; ^2^ Monash University, Melbourne, VIC Australia; ^3^ University of Sydney, Sydney, NSW Australia; ^4^ Dementia Alliance International, Sydney, NSW Australia; ^5^ University of New South Wales, Sydney, NSW Australia; ^6^ University of Wollongong, Wollongong, NSW Australia; ^7^ University of New South Wales (UNSW), Sydney, NSW Australia

## Abstract

**Background:**

Behaviour support plans (BSPs) for people in residential aged care (RAC) were mandated nationally in 2019 for those who require, or may require, restrictive practices as part of their care. The legislation aims to reduce and potentially eliminate restrictive practices: long‐standing problems of their inappropriate use were highlighted by the Royal Commission into Aged Care Quality and Safety (2018).

Many people living with dementia will be impacted: up to 90% experience changed behaviours; 54% of people in RACs have dementia; and approximately 20% experience cognitive decline without a diagnosis.

There is no research examining how BSPs are implemented in practice since legislation was introduced. Our exploratory pilot study encompasses behaviour support policies, BSPs and staff views to identify practices, themes, barriers and enablers. This paper focuses on the content of BSPs.

**Method:**

RAC providers were recruited via professional networks and requested to include two sites, submitting three BSPs for residents from each site. Age, gender and diagnosis from redacted plans were extracted, with legislation used as a framework to extract further content for analysis, including behaviours and non‐pharmacological strategies prescribed.

**Result:**

Preliminary results are presented with analysis continuing. Seven providers (24 sites) across eastern Australian states submitted sixty BSPs, which varied in format, content, and length (range 1‐27 pages). Residents were mostly female (n = 41; 68%), aged 84 years (mean)(± 8.0 years; range 65‐98), and according to BSPs, had dementia/cognitive impairment (n = 57; 95%). Agitation and ‘wandering’ were the most frequently reported behaviours. Restrictive practices use was common (n = 46, 77%), including chemical restraint (n = 14; 30%), environmental secure unit restraint (n = 14; 30%), or both chemical and environmental restraint (n = 15; 33%).

Variability in number and specificity of behaviour support strategies prescribed was evident: some plans included 4‐5 generic strategies, such as offering drinks/snacks; others detailed multiple strategies, including specific times of need and clear resident support preferences.

**Conclusion:**

Our study suggests that implementation of behaviour support legislation as reflected in care planning documentation is inconsistent, in terms of what constitutes behaviour and how best to support residents. Positive framing of behaviour support is limited. Guidance and education for the sector are urgently indicated.

